# Physical Activity of Children with Physical Disabilities: Associations with Environmental and Behavioral Variables at Home and School

**DOI:** 10.3390/ijerph16081394

**Published:** 2019-04-17

**Authors:** Cindy Sit, Ru Li, Thomas L. McKenzie, Ester Cerin, Stephen Wong, Raymond Sum, Elean Leung

**Affiliations:** 1 Department of Sports Science and Physical Education, The Chinese University of Hong Kong, Shatin, New Territories, Hong Kong; lirutracy@szu.edu.cn (R.L.); hsswong@cuhk.edu.hk (S.W.); kwsum@cuhk.edu.h (R.S.); 2 Department of Physical Education, Shenzhen University, Shenzhen, China; 3 School of Exercise and Nutritional Sciences, San Diego State University, San Diego, CA 92182, USA; tmckenzi@sdsu.edu; 4 Mary MacKillop Institute for Health Research, Australian Catholic University, Melbourne 3000, Australia; ester.cerin@acu.edu.au; 5 School of Public Health, The University of Hong Kong, Hong Kong; 6 Physical Education Unit, The Chinese University of Hong Kong, Shatin, New Territories, Hong Kong; elean@cuhk.edu.hk

**Keywords:** youth, sedentary behavior, observation, family, peers

## Abstract

The purpose of this study was to examine the physical activity (PA) of children with physical disabilities (PD) and its associated environmental and behavioral factors at home and at school. One hundred and forty-seven Hong Kong children (mean age = 13.5 ± 2.5 years) with PD from three special schools participated. We used BEACHES (Behaviors of Eating and Activity for Children’s Health: Evaluation System) to assess their PA and associated variables at home (before dinner) and during four school settings (before classes, recess, lunch breaks, after classes) on four school days. Overall, the children were typically inactive and spent little time in moderate-to-vigorous physical activity (MVPA), range = 6.3% to 17.0% across settings. At home, children were more active when fathers were present (*p* < 0.001). At school, prompts to be active contributed to their MVPA% before classes (*p* < 0.01) and during recess and lunch breaks (both *p* < 0.001). The presence of a child’s mother was positively associated with MVPA% before classes (*p* < 0.001) and the presence of other children was associated with MVPA% during recess and lunch breaks (both *p* < 0.05). With children with PD accruing small amounts of MVPA in both home and school settings, multifaceted interventions reflecting both contextual and personal factors should be considered in order to increase the health-enhancing PA of this population.

## 1. Introduction

With the high prevalence of sedentary living and its association with obesity and other related health problems, the promotion of physical activity (PA) has become a global public health priority [1]. As a result, there has been an increasing number of studies on PA and associated environmental conditions, most of which have focused on typically developing children and adults without disabling conditions. Meanwhile, nearly 15% of the world’s population (equivalent to one billion people) live with a disability, with about 95 million of them being children [2]. An estimated 320,500 people in Hong Kong (4.5% of the population) have a physical disability (PD) [3], but rarely is their PA studied [4].

Current health guidelines recommend that children engage in at least 60 min of moderate-to-vigorous physical activity (MVPA) daily [5], but children with PD fall far short of this goal and are thus insufficiently active for health purposes [6,7]. To design effective interventions and create appropriate PA programs for this population, there is a need to understand factors that influence their PA engagement. Reviews of correlates have shown that environmental factors such as activity area size and family and peer social support affect the amount and intensity of PA among children with PD [4,8]. A limitation to previous studies has been the assumption that environmental influences on PA are invariant across different contexts, and Atkin et al. [9] proposed that future studies consider the specific contexts where PA takes place. Understanding potentially modifiable contextual conditions that are associated with PA requires an assessment of environmental characteristics such as location, people present, time of day or week, and activity type. Doing so would advance our knowledge regarding the uniqueness of factors impacting of PA within different contexts [8].

Homes and schools are the primary places for children to accrue PA, but these locations were assigned only “D−” and “C” grades, respectively, on Hong Kong’s 2018 Report Card on Physical Activity for Children and Youth [10]. Such low scores indicate that both environments fall short in providing ample PA opportunities for children. Subsequently it is important to assess the activity levels of children with PD within these settings to determine factors that influence their active engagement [11]. Accelerometry can be used to measure PA among children with PD [12], but this method prohibits the assessment of concurrent and highly variable contextual conditions that may affect children’s PA. Meanwhile, direct observation methods have the ability to provide detailed information on both the physical and social contexts in which the activity occurs [11,13] and they have been used previously to study PA and associated conditions among children with disabilities [14,15,16]. BEACHES (Behaviors of Eating and Activity for Children’s Health: Evaluation System) [17], for example, was designed specifically to assess children’s PA and eating behaviors while simultaneously recording associated physical and social environmental conditions in both home and school settings.

BEACHES has been validated and used to assess the PA of both children with typical development [18] and those with PD [16]. In a preliminary study, we used BEACHES to assess Hong Kong children with PD and found that they spent most of their time at home and at school being physically inactive [19]. Although important and the first of its kind to assess the PA environments of children with PD, that study included only 35 children from a single school and thus had limited generalizability. The current study, which expands that initial investigation to include 147 children from three of the seven special schools for children with PD in Hong Kong, permits a more sophisticated data analyses and increases the generalizability of the results. The purpose of the study, therefore, was to examine PA and its associated factors among children with PD at home and at school. Specifically, we (a) assessed the PA of children with PD during leisure time at home (before dinner) and at school (recess, lunch breaks, and before and after classes), and (b) examined the occurrence of context-specific physical and social characteristics and their associations with PA in these five settings.

## 2. Materials and Methods

### 2.1. Participants

Data for this study of 147 Hong Kong Chinese students (boys, *n* = 90; girls, *n* = 57), aged 13.5 ± 2.5 years were collected during spring of the 2015–2016 school year. They were enrolled in three of the seven special schools in Hong Kong for children with PD. Inclusion criteria were boys and girls in grades 4 to 12 attending special schools for children with PD. Exclusion criteria included those children with a disease that affected their cognitive functioning. Demographic information such as gender and grade and mobility levels were collected directly from the school records. Written informed parental consent and child assent were obtained for each participant.

The study complied with the principles of the Declaration of Helsinki and was approved by the Joint Chinese University of Hong Kong—New Territories East Cluster Clinical Research Ethics Committee (reference number CRE-2014.060).

### 2.2. Measures

#### 2.2.1. Observation System

BEACHES documents children’s PA and eating behaviors while simultaneously recording associated environmental characteristics and events in both home and school settings [17]. The instrument has been validated and used with children with typical development [18,20], and its activity codes have been validated via accelerometry with a small sample of children with PD in both home and school settings [16].

Trained observers used BEACHES to code seven dimensions: (a) *Child PA Level* (lying down, sitting, standing, walking/moderate, or vigorous); (b) *Child Location* (inside home/residence, outside home/residence); (c) *People Present* (i.e., mother, father, another child, and another adult in the child’s immediate environment); (d) *Behavior Motivated* (events related to motivating/influencing the target child to directly engage in PA or sedentary behavior or to ingest food; (e) *Motivator* (none, mother, father, other child, and other adult); (f) *Views Media* (whether or not the child engaged in media-related activity such as TV viewing); and (g) *Eats* (whether or not the child ingested food). 

#### 2.2.2. Observation Procedures

Observer training and maintenance. Twenty observers were trained to collect data via the standard BEACHES observer training protocol. It consisted of observers first memorizing the behavioral dimensions and subcategories and then learning how to enter data. Video examples and role playing were used during classroom sessions and this was followed by both video and field practice sessions. Training for each observer continued until he/she exceeded an inter-observer agreement (IOA) score of 85% on a criterion video. Additionally, approximately 10% of observations during data collection were completed simultaneously by two independent observers. Results of these paired observations indicated good to excellent interobserver reliability for all seven BEACHES dimensions (ranging from 89% to 97% IOA), with a mean kappa ranging from 0.74 to 0.95.

Pacing of observations. Observers, paced by voice prompts from digital players, used alternating observe-record intervals to focus directly on the target child for 15 s and had up to 15 s to enter data. The first three dimensions (child PA level, child location, people present) were scored using momentary time sampling (i.e., event was occurring at the end (at the moment) the “observe” interval ended). The remaining four dimensions (behavior motivated, motivator, views media, eats) were scored using partial-interval time sampling (i.e., event occurred at any time during the “observe” interval) resulting in the outcome being “% of intervals”, not “% of the time”.

Observations at home. Similar to our preliminary study [19], children were observed at their home/residence on the same four days they were observed at school. Observation periods were planned to be 60 min in length [21] between the time the child arrived at home/residence and the start of dinner.

Observations at school. Children were observed during four typical unstructured times/sessions that occurred at their schools (i.e., before classes started, and during recess, lunch breaks, and after classes). Observations were scheduled to take place on normal school days with a different day identified each week over four consecutive weeks. The length of observation sessions was planned (i.e., recess, 30-min; lunch breaks, 30-min; before classes, 30-min before official start of the school day; and after classes, 60-min after official end of school). Nonetheless, actual observed time depended on each child’s personal schedule and last-minute modifications to a school timetable (e.g., a child arriving late to school or an after-school session being shortened by staff).

### 2.3. Data Analysis

Descriptive statistics, including the mean proportion of observation intervals (or time) were used to summarize the observed PA levels (i.e., lying down, sitting, standing, walking/moderate, and vigorous) and the environmental and behavioral characteristics by setting. Moderate-to-vigorous physical activity (MVPA) was obtained by summing the walking/moderate and vigorous codes. A series of linear mixed models (LMMs) were performed to assess the association of the environmental and behavioral variables with MVPA % after adjusting for confounders (gender, grade levels (primary, junior, senior), and mobility level (walking with used assistive devices, walked unassisted)). LMM was selected because it can accommodate incomplete datasets with repeated measures (i.e., 4 measurement days) while considering school clustering effects. For each LMM, measurement days (*n* = 4) were used as a repeated measures variable. School was considered as a random effect and the demographic variables and environmental characteristics were fixed effects in the models. Statistical analyses were performed using SPSS 23.0 (IBM Corp, Armonk, NY, US) and a significance level of 0.05 was adopted.

## 3. Results

### 3.1. Characteristics of Participants

Table 1 describes the gender, grade level and mobility level of the participants. Three of 150 eligible students that initially agreed to participate withdrew, leaving an analytic sample of 147 students (Mean Age = 13.5 years; SD = 2.5 years) that were distributed evenly across primary, junior and senior grade levels. Most participants were boys (*n* = 90, 61.2%), and nearly half (*n* = 68, 46.3%) used assistive devices to walk.

### 3.2. Descriptive Statistics for the Seven Dimensions at Home and School Settings

Table 2 presents the mean proportion of intervals (and calculated minutes) that the children spent in various activity levels at home and at school. Mean observed time among settings ranged from 15.0 to 54.3 min, with children being inactive most of that time (ranging from 60.7% to 82% across settings). They were physically active only a small proportion of time in any setting (MVPA range = 6.3% to 17.0%; vigorous range = 0.8% to 6.7%).

Table 2 also shows that children spent most of their observed time at home being indoors (79.0%) and inactive (82%), and rarely alone in the area (11%). They were frequently accompanied by another child (56.4% of intervals) and a non-parent adult (63.6% of intervals), with mothers and fathers present 28.2% and 7.1% of intervals, respectively. Nearly half their intervals (49.0%) included viewing electronic media and they rarely ingested food (5.6% of intervals) or were prompted or reinforced to engage in either PA or sedentary behavior (about 5% of intervals).

In contrast, at school the children spent nearly all their observed time outdoors. They were rarely alone (about 2% of the time) and typically shared their setting with another child (about 94% of the time) and an adult such as teacher or playground supervisor (about 90% of the time).

Except for during the after-class sessions at school, the children were rarely motivated (i.e., prompted or reinforced) to engage in either physically active or sedentary (i.e., lie down or sit) behavior. Rates for motivating PA and sedentary behavior were similar among the five settings (e.g., at home, 5.3% vs. 5.1% of observed intervals). Compared to the home setting, children’s engagement with electronic media at school was low (range = 2.9% to 16.5% of intervals across settings). Rarely did children ingest food during the observed periods (range = 0.6% to 5.6% of intervals across settings).

### 3.3. Contextual Associations with Physical Activity

Table 3 shows the results of using LMMs to examine the effects of selected environmental and behavioral variables on MVPA% across settings while adjusting for gender, grade and mobility levels. MVPA% at home did not differ significantly between those using assistive devices and who walked unassisted. At school, however, those who walked unassisted had greater MVPA% in all four settings: before classes, *F*(1, 103.91) = 6.00, *p* < 0.05); recess, *F*(1, 143.18) = 52.13, *p* < 0.001; lunch breaks, *F*(1, 136.43) = 36.57, *p* < 0.001; and after classes, *F*(1, 80.97) = 10.77, *p* < 0.01).

Specific environmental and behavioral factors were associated with PA in the settings. At school, for example, prompts to be active from others contributed significantly to children’s MVPA% before classes and during recess and lunch breaks. A one-unit increase in motivation prompts/reinforcers to be active from others was associated with a 0.40% increase in MVPA% before classes (*b* = 0.40, 95% CI = (0.17, 0.64), *p* = 0.007), a 0.46% increase during recess (*b* = 0.46, 95% CI = (0.33, 0.59), *p* < 0.001), and a 0.54% increase during lunch breaks (*b* = 0.54, 95% CI = (0.43, 0.65), *p* < 0.001). Additionally, motivation from another child was positively associated with increased MVPA% during both recess (*b* = 0.23, 95% CI = (0.02, 0.43), *p* = 0.046) and lunch breaks (*b* = 0.13, 95% CI = (0.03, 0.23), *p* = 0.048). Also, prompts to engage in sedentary behavior by others was negatively associated with MVPA% engagement during after classes (*b* = −0.37, 95% CI = (−0.58, −0.16), *p* = 0.001).

The presence of parents was also related to children’s PA engagement. Specifically, the presence of the mother was positively associated with a child’s MVPA% before classes at school (*b* = 0.74, 95% CI = (0.51, 0.96), *p* < 0.001) and at home before dinner children were more likely to be physically active when their fathers were present (*b* = 0.27, 95% CI = (0.14, 0.39), *p* < 0.001).

## 4. Discussion

Physical activity is essential for children’s current and future health and development and home and school settings are primary locations where they can accrue it. This investigation extends previous direct observation studies by specifically assessing PA and associated contextual characteristics among children with PD at home before dinner and in four distinct leisure time settings at school. Previous studies of children’s PA during leisure time at school focused primarily on recess periods [15,16,22] and other unstructured times at school such as before and after classes and during lunch breaks have been overlooked.

Consistent with previous studies [15,16], children with PD were in general physically inactive across all settings (ranging from 60.7% to 82% of observed time). Recess and after-class sessions at schools provided slightly higher activity rates than other settings. At home, children spent most of their time indoors, often viewing electronic media. Similar to previous BEACHES studies [20,23], the presence of others who provided prompts for activity was highly associated with PA in the school settings. Congruent with other studies of children with PD [24,25], being motivated (i.e., prompted or reinforced) to be physically active was a significant predictor of children’s PA before classes and during recess and lunch breaks. Unlike studies that frequently report boys to be more physically active than girls [5], gender differences in PA were not found in any of the five settings studied.

Interestingly, the presence of the mother was significantly associated with the children’s MVPA before classes at school. In many Hong Kong special schools, parents (usually mothers) take their children to school and engage with them before the start of classes. During recess and lunch breaks, other children served as a source of motivation by providing PA prompts and this was also associated with increased MVPA. The positive effect of peer support on the PA of children with typical development has been noted previously [26,27], and the current study found that interactions with peers directly affected the activity-related behavior of children with PD in the immediate environment. For example, motivational prompts to be active from peers were related to increased MVPA% at recess and during lunch breaks. As well, prompts to be sedentary (e.g., view media) were negatively associated with PA after classes at school. This may be because most after-class activities at school were of a sedentary nature, including engaging in drawing, academic tutorials, computer activities, and watching movies. Finding that the presence of fathers to be related to the children’s MVPA at home concurs with previous studies that found fathers play a strong role in influencing their children’s PA [28,29,30].

In general, the contextual correlates of PA varied across the home and school settings, and this was related mainly to differences in the unique role of motivators in the different locations. The presence of mothers influenced their children’s PA before classes at school; prompts for PA engagement by peers were associated with PA during recess and lunch breaks; and the presence of fathers influenced children’s activity levels at home. The important role of social support from peers and adults in promoting children’s PA has been found using different methods in previous research [8], and our use of micro-level, simultaneous direct observations of PA and verbal and non-verbal interactions in the current study further verified this notion. Additionally, this study extended the reach of the previous social support findings to include children with PD in both home and school settings.

Study strengths include reliable observers, a large sample size, and assessments during four school settings and at home before dinner when most family members were likely to be present. Nonetheless, the study has limitations. These include that all participants were ambulatory and attending Hong Kong schools that were designed specifically for children with PD. To promote generalizability, similar studies should widen the range of disability to include non-ambulatory children as well as students enrolled in mainstreamed schools and from other countries. Additionally, as a real-world investigation, study participants were not always present to be observed throughout each of the five planned sessions (i.e., before classes, recess, lunch breaks, and after classes at school and before dinner at home). Observations did not include weekends, and children being present at school before the start of classes and after school was hampered by transportation challenges and parent schedules. Future research should address the aforementioned limitations and examine other disability groups (e.g., children with intellectual disabilities) to help advance our understanding of children’s PA in both settings.

Despite these limitations, the study findings highlight the limited amounts of PA that ambulatory children with PD accrue both at home and at school. Children with PD are found to spend only 0.44 h in MVPA daily, and only 25% of them are able to meet the recommended level of MVPA [31]. Interventions are needed, and planning for them should consider the importance of social support for PA, including the presence of parents and prompts from family and peers at school.

## 5. Conclusions

This study is one of the very few to systematically document the simultaneous occurrence of PA and its associated contextual characteristics among children with PD during leisure time both at home and at school. These children with PD accrued little MVPA during leisure time periods both at home (about 6.3% of observed time) and at school (about 15.7% of observed time). Modifications within both the home and school settings have potential for increasing the health producing PA of children with PD.

## Figures and Tables

**Table 1:** Gender and grade and mobility levels of participants (total *n* = 147).

Variable	Number (%)
Gender	
Boys	90 (61.2)
Girls	57 (38.8)
Grade level	
Primary (Grades 4–6)	48 (32.7)
Junior (Grades 7–9)	48 (32.7)
Senior (Grades 10–12)	51 (34.7)
Mobility level	
Walked unassisted	79 (53.7)
Walked used assistive devices	68 (46.3)

**Table 2:** Mean data for the seven dimensions observed in home and school settings.

Category	Home	School			
	Before Dinner *n* = 62	Before Classes *n* = 42	Recess *n* = 146	Lunch Breaks *n* = 147	After Classes *n* = 80
	Mean Min. = 46.6	Mean Min. = 15.0	Mean Min. = 24.2	Mean Min. = 28.3	Mean Min. = 54.3
**Physical activity level**					
Lying down % (min)	1.3 (0.6)	1.4 (0.2)	0.4 (0.1)	1.3 (0.4)	0.6 (0.3)
Sitting % (min)	80.7 (37.6)	64.6 (9.7)	60.3 (14.6)	63.0 (17.8)	64.0 (34.7)
Standing % (min)	11.7 (5.5)	21.2 (3.2)	22.4 (5.4)	21.6 (6.1)	18.7 (10.1)
Walking % (min)	5.4 (2.5)	11.9 (1.8)	15.6 (3.8)	12.4 (3.5)	10.0 (5.4)
Vigorous % (min)	0.8 (0.4)	1.0 (0.1)	1.4 (0.3)	1.7 (0.5)	6.7 (3.6)
MVPA % (min)	6.3 (2.9)	12.8 (1.9)	17.0 (4.1)	14.1 (4.0)	16.8 (9.1)
**Location**					
Inside % (min)	79.0 (36.8)	0.3 (0.0)	0.0 (0.0)	0.5 (0.1)	3.2 (1.7)
Outside % (min)	21.0 (9.8)	99.7 (15.0)	100.0 (24.2)	99.5 (28.2)	96.8 (52.6)
**People present**					
Alone % (min)	11.3 (5.3)	2.1 (0.3)	1.9 (0.5)	1.9 (0.5)	1.1 (0.6)
Mother % (min)	28.1 (13.1)	0.5 (0.1)	0.4 (0.1)	0.6 (0.2)	2.7 (1.5)
Father % (min)	7.1 (3.3)	0.0 (0.0)	0.0 (0.0)	0.1 (0.0)	0.0 (0.0)
Other child % (min)	56.4 (26.3)	95.5 (14.3)	95.1 (23.0)	94.9 (26.9)	91.8 (49.8)
Other adult % (min)	63.6 (29.6)	87.4 (13.1)	87.1 (21.1)	86.3 (24.4)	95.1 (51.6)
**Behavior motivated** ^†^					
None %	84.4	89.5	85.1	84.6	71.9
Physical activity %	5.3	5.2	5.6	5.2	14.0
Sedentary behavior %	5.1	5.0	6.7	7.6	11.6
Eating %	1.3	0.0	1.7	1.2	0.3
Not eating %	0.3	0.0	0.0	0.0	0.0
Media %	3.4	0.3	0.8	1.3	2.2
No media %	0.2	0.0	0.1	0.1	0.0
**Motivator** ^†^					
None %	84.7	89.6	85.5	84.8	71.8
Mother %	2.6	0.2	0.3	0.4	0.9
Father %	0.2	0.0	0.0	0.0	0.0
Other child %	2.8	0.9	3.7	4.9	1.7
Other adult %	9.7	9.3	10.5	9.9	25.6
**Views media** ^†^					
No %	51.0	97.1	92.2	86.9	83.5
Yes %	49.0	2.9	7.8	13.1	16.5
**Ingests food** ^†^					
No %	94.4	99.4	95.2	96.4	99.0
Yes %	5.6	0.6	4.8	3.6	1.0

MVPA = walking/moderate + vigorous; Time estimates cannot be determined for categories ^†^ using partial interval recording.

**Table 3:** Associations between selected variables and MVPA% in different settings, adjusting for child gender and grade and mobility levels.

Setting	MVPA%			
	Coefficient	SE	95% CI	*p*
**Before dinner (home)**				
Intercept (ICC = 0.01)	1.88	3.34	−4.80 to 8.56	0.62
Gender (boys)	2.88	2.50	−2.16 to 7.92	0.20
Grade level (4–6)	2.74	3.10	−3.47 to 8.95	0.69
Grade level (7–9)	0.05	3.17	−6.31 to 6.41	0.85
Mobility (walked unassisted)	1.02	2.24	−3.50 to 5.53	0.54
Father present %	0.27	0.06	0.14 to 0.39	<0.001
**Before classes**				
Intercept (ICC = 0.01)	16.11	7.20	1.92 to 30.30	0.96
Gender (boys)	−4.02	2.45	−8.89 to 0.85	0.08
Grade level (4–6)	3.34	2.96	−2.53 to 9.22	0.24
Grade level (7–9)	3.53	3.10	−2.62 to 9.68	0.18
Mobility (walked unassisted)	6.20	2.53	1.18 to 11.22	0.02
Mother present %	0.74	0.11	0.51 to 0.96	<0.001
Physical activity motivated %	0.40	0.12	0.17 to 0.64	0.007
**Recess**				
Intercept (ICC = 0.04)	5.62	0.67	0.34 to 10.89	0.26
Gender (boys)	0.52	2.31	−4.04 to 5.09	0.64
Grade level (4–6)	−0.55	2.75	−5.98 to 4.88	0.99
Grade level (7–9)	1.31	2.73	−4.09 to 6.71	0.60
Mobility (walked unassisted)	16.42	2.27	11.93 to 20.92	<0.001
Physical activity motivated %	0.46	0.07	0.33 to 0.59	<0.001
Other child as motivator %	0.23	0.11	0.02 to 0.43	0.046
**Lunch breaks**				
Intercept (ICC = 0.11)	6.82	2.21	2.45 to 11.19	0.76
Gender (boys)	−2.43	1.91	−6.21 to 1.36	0.23
Grade level (4–6)	−0.05	2.28	−4.56 to 4.46	0.57
Grade level (7–9)	1.27	2.26	−3.20 to 5.75	0.79
Mobility (walked unassisted)	11.39	1.88	7.67 to 15.12	<0.001
Physical activity motivated %	0.54	0.06	0.43 to 0.65	<0.001
Other child as motivator %	0.13	0.05	0.03 to 0.23	0.048
**After classes**				
Intercept (ICC = 0.06)	18.58	5.95	6.74 to 30.41	0.002
Gender (boys)	−7.12	5.34	−17.73 to 3.49	0.19
Grade level (4–6)	−1.75	6.12	−13.95 to 10.44	0.78
Grade level (7–9)	−8.59	5.81	−20.16 to 2.98	0.14
Mobility (walked unassisted)	16.33	4.98	6.43 to 26.24	0.002
Sedentary behavior motivated %	−0.37	0.10	−0.58 to −0.16	0.001

MVPA% = percentage of time spent in moderate-to-vigorous physical activity; SE = standard error; ICC = Intraclass correlation coefficient. Findings are presented as regression coefficients, standard error, and 95% confidence interval (95% CI) and were based on linear mixed model with school as random effects and observation days as a repeated-measure variable. Significant *p* values are in bold. Reference group: gender (0 = girls), grade level (0 = 10–12), mobility (0 = walked with assistive device).
